# Outcomes of community-based differentiated models of multi-month dispensing of antiretroviral medication among stable HIV-infected patients in Lesotho: a cluster randomised non-inferiority trial protocol

**DOI:** 10.1186/s12889-018-5961-0

**Published:** 2018-08-29

**Authors:** I. O. Faturiyele, T. Appolinare, N. Ngorima-Mabhena, G. Fatti, I. Tshabalala, V. J. Tukei, P. T. Pisa

**Affiliations:** 1 Right to Care/EQUIP, Maseru, Lesotho; 2 EGPAF, Washington, DC USA; 3 Kheth’Impilo /EQUIP, Harare, Zimbabwe; 4 Kheth’Impilo AIDS Free Living, Cape Town, South Africa; 5 Division of Epidemiology and Biostatistics, Department of Global Health, Faculty of Medicine and Health Sciences, Stellenbosch University, Stellenbosch, South Africa; 6 Ministry of Health, Maseru, Lesotho; 7 EGPAF, Maseru, Lesotho; 8 Right to Care/EQUIP, Centurion, South Africa

**Keywords:** Differentiated models of care, Antiretroviral therapy, HIV, Retention, Virologic suppression, Cost-effectiveness, Lesotho

## Abstract

**Background:**

Current World Health Organization (WHO) guidelines recommend early initiation of HIV positive patients on antiretroviral therapy (ART) irrespective of their clinical or immunological status known as the test and start approach. Lesotho, like many other countries introduced this approach in 2016 as a strategy to reach epidemic control. There will be rapidly growing number of HIV-infected individuals initiating treatment leading to practical challenges on health systems such as congestion, long waiting time for patients and limited time to provide quality services to patients. Differentiated models of ART delivery is an innovative solution that helps to increase access to care, while reducing the burden on existing health systems. Ultimately this model will help to achieve retention and viral suppression. We describe a demonstration study designed to evaluate a community-based differentiated model of multi-month dispensing (MMD) approaches of ART among stable HIV patients in Lesotho.

**Methods:**

This study will be a three-arm cluster randomised trial, which will enrol approximately 5760 HIV-infected individuals who are stable on ART in 30 selected clusters. The clusters, which are health facilities, will be randomly assigned into the following differentiated model of care arms: (i) 3 monthly ART supply at facilities (Control), (ii) 3 monthly ART supply through community ART groups (CAGs) and (iii) 6 monthly ART supply through community ART distribution points (CAD). Primary outcomes are retention in care and virologic suppression, and secondary outcomes include feasibility and cost effectiveness.

**Discussion:**

Important lessons will be learnt to allow for improved implementation of such demonstration projects, including various needs for reliable supply of medication, access to quality clinical data including access to viral loads (VLs) results, frameworks to support lay worker cadre, involvement of community stakeholders, and reliable data systems including records of key indicators. MMD will have positive implications including improved retention, virologic suppression, convenience and access to medication.

**Trial registration:**

ClinicalTrials.gov Identifier: NCT03438370. Accepted on 16 February 2018.

**Electronic supplementary material:**

The online version of this article (10.1186/s12889-018-5961-0) contains supplementary material, which is available to authorized users.

## Background

Lesotho is undergoing a significant health transition characterised by high burden of infectious diseases such as HIV/AIDS, tuberculosis and an emergence of non-communicable diseases [1]. Among adults, HIV prevalence in Lesotho is reported to be 25% and 53% of adults are receiving antiretroviral treatment (ART) [2]. An estimated 310,000 people were living with HIV and 18,000 died from AIDS related issues in 2015 [3]. PEPFAR adopted the UNAIDS 90–90-90 targets and the new WHO guidelines on when to initiate ART [4]. The WHO guidelines recommend ART initiation immediately after testing positive, regardless of CD4 count, with lifelong continuation to warrant and improve individual ‘s health and reduce HIV transmission [5]. To realize the full benefits of this approach, high levels of adherence and retention are needed. In low to middle income countries (LMICs) including Lesotho, long waiting times in ART clinics, cost of travel to clinics and other life commitments are known to disrupt and reduce the adherence and retention levels. A need for monthly dispensing and/or systems that require multiple separate visits for refills and clinical evaluations can raise significant challenges for people living with HIV (PLHIV) and result in treatment interruptions or complete disengagement from care [6]. Hence, extending of interval ART refills may improve outcomes, if supply can be guaranteed for up to six months for stable patients in low resourced settings.

Currently, many LMICs including Lesotho dispense ART monthly which places demands on the health system and can lead to suboptimal adherence and disengagement in care due to the time and cost of frequent visits to clinic. There is need to implement novel strategies for ART dispensing to enable health systems cope with the ever-increasing number of HIV patients needing care and treatment. A gap exists in allowing stable patients to have an adequate and longer-term supply of ART especially if this does not compromise outcomes of interest including retention and viral load (VL) suppression.

Community based models of care i.e. CAGs are postulated to further strengthen adherence, retention and viral suppression. These models give peer support among patients. CAGs have been successfully implemented in Lesotho and introducing MMD into them can have the same intended outcomes as facility-based interventions, whilst additionally decongesting and decentralising facilities.

Potential benefits for this approach includes (i) higher adherence to ART and retention in care, (ii) reduced per-patient cost of providing ART, by reducing the number of clinic visits required, (iii) decongestion of clinics to allow for increased capacity to manage patients newly diagnosed with HIV, those with infectious complications, treatment failure, and other co-morbidities and (iv) decreased waiting time and improved efficiency at clinics allowing for improved quality of care and patient satisfaction [7, 8].

Limited data are available from LMICs on multi-month scripting and dispensing and on studies using community-based models of care. A review of the literature reveals a lack of randomized studies of different ART dispensing intervals. An abstract presented at the Conference on Retroviruses and Opportunistic Infections, 2016 using Zambian data, suggested that requirements for monthly dispensing and/or systems that require multiple separate visits for refills and clinician evaluation can raise significant challenges for people living with HIV and result in treatment interruptions or complete disengagement from care [6].

This study’s primary objectives are (i) to determine if stable patients receiving 3 monthly dispensing of ART within CAGs have non-inferior retention in care and viral load suppression than stable ART patients receiving 3 monthly dispensing at health facilities after 12 and 24 months, (ii) 6 monthly dispensing of ART within the community from a health care worker have non-inferior retention in care than stable ART patients receiving 3 monthly dispensing at health facilities after 12 and 24 months and (iii) 6 monthly dispensing of ART within the community from a healthcare worker have non-inferior retention in care than stable ART patients receiving 3 monthly dispensing within CAGs after 12 and 24 months. The secondary objectives are to compare cost outcomes (cost per patient retained and cost per patient retained with viral suppression) between the three models, investigate the acceptability of 3 and 6 monthly community-based ART distribution among patients and service providers and to assess if there are measurable gains in clinic productivity realized following the introduction of community based MMD of ART.

## Methods/design

### Setting

This study will include 30 health facilities (clusters) purposely selected from Maseru, Mafeteng and Mohale’s Hoek districts. These districts have been purposively selected due to (i) high numbers of patients on ART, (ii) has established and have since prioritised CAGs (iii) ability to perform VL determination as part of routine care. Facilities are to be included in the study if they have implemented or have systems in place for CAGs or community ART dispensing (CAD) through a healthcare worker, CAGs and CAD points are feasible within the health facilities community, health facility routine data collection and systems are in place, completeness of medical records, ability to perform VL determination as part of routine care, existing drug supply chain procedures for multi-month dispensing, and a large enough population to enrol stable ART patients.

### Study design

**Fig. 1:**
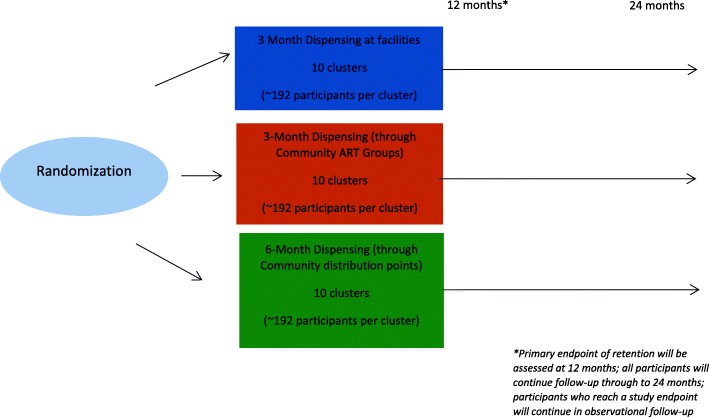
Multi-Month Community Dispensing of ART: Examining the Role of Differentiated models of ART Supply on Retention and Virologic Suppression

The study will be a pragmatic, three arm, parallel, cluster-randomized non-inferiority design. The thirty facilities (clusters) will be randomly assigned to three arms (Fig. 1) with patients receiving (i) 3MF- 3 monthly ART supply at facilities (Control), (ii) 3MC - 3 monthly ART supply through community ART groups (CAGs) and (iii) 6MCD - 6 monthly ART supply through community distribution points (CAD). Included facilities will be randomized, with stratification by whether the facility is in a rural or urban setting according to the Lesotho Bureau of Statistics’ data. After this process, each arm will, contain 10 facilities.

### Study population and eligibility

This study will be conducted among approximately 5760 HIV-infected individuals who are stable on ART in 30 selected clusters. The eligibility criteria of participants are tabulated in Table 1.

**Table 1:** Eligibility criteria for participants

To be included are those:	
(i) 18 years of age or older and willing to provide written informed consent,	
(ii) willing to participate in the MMD model (arm) that the patient’s study cluster has been randomized to	
(iii) On ART ≥ 6 months with no periods of defaulting on treatment since the last VL result (ART default defined as missing 7 or more consecutive days of ART	
(iv) On first-line ART regimen (substitutions within the first-line regimen prior to the last VL test are permissible)	
(v) No ARV drug substitutions since the last VL result < 1,000 copies/ml and plasma or dried-blood spot VL < 1,000 copies/ml in a patient who has been on first-line ART for at least 6 months, with VL drawn within last 12 months of enrolment while patient is receiving ART	
To be excluded are those:	
(i) on any other ART line regimen	
(ii) with co-morbidities requiring more frequent facility visits	
(iii) ART substitutions since last VL test (iv) diagnosed with a WHO clinical stage 3 or 4 condition within the past 3 months	
(iv) pregnant or less than 12 months postpartum and breastfeeding mothers	
(v) participating in another study that involves dispensing interval, adherence, or retention or involves receiving medications	

### Study procedures: recruitment, screening and enrolment processes

Recruitment methods for this study will be comparable across clusters (health facilities) and are expected to rely on active identification and referral of stable patients, as defined in the inclusion criteria above through relevant study staff. Facilities will be randomized to either of the 3 arms and individuals will receive their first drug supplies to which the cluster has been randomly assigned at enrolment. However, after first dispensing of drugs at the facility, those randomized to the arm to which patients are to receive 3 and 6 monthly ART supply through CAGs and CAD will respectively receive their next ART refills through these community approaches and platforms. Only those randomized to the 3 monthly ART-supply at facilities will receive their next ART refill within facilities.

The process of consenting participant’s will be fully documented, and only individuals who are able to demonstrate understanding will be asked to provide written informed consent for study screening and enrolment.

All patients potentially eligible for the study would also be eligible for a VL test per the national ART guidelines if they had not previously had one in the last 12 months. Once the VL result is back, they will be re-screened for study eligibility when they return for their next routine visit. After enrolment into study, co-enrolment in other studies will not be allowed given the likelihood that this could interfere with the primary study endpoint of retention.

### Participant retention and withdrawal or termination from the study

As retention in care is a primary outcome for the study, once an individual is enrolled, there will be no additional efforts applied towards retaining them except for standard of care counselling and routine retention support offered by sites as per national guidelines. Study interactions with participants will be minimized through use of existing medical records to track patient outcomes. We will obtain informed consent to contact people after completion of the study to perform qualitative interviews and surveys of acceptability, cost and quality of life measures. Regardless of the participant retention procedures referenced above, individuals may voluntarily withdraw from the study.

### Study intervention: differentiated models of care for MMD of ART

#### Three month dispensing model at health facilities

Providers at facilities randomized to three-month dispensing will be expected to provide all enrolled patients with a 3-month supply of ART. All other aspects of care will be as standard of care for the enrolling clinic, though routine clinic visits will occur every 90 days instead of the standard of care interval. Information about ideal storage conditions for ART will be provided by the clinic.

#### Community ART group (CAG) model (3-month supply of drug)

Study participants enrolled at facilities in this arm will join new or previously existing CAGs or will already be members of a CAG. Their first 3-month supply of drugs will be at enrolment facility, whilst the next ART refills will be through CAGs. The CAG will consist of 4–12 participants, who live in a similar geographic location and who all attend the same health care facility. The members’ appointments will be synchronized to ensure their scheduled clinic visits will be on the same day. The CAG will nominate a CAG leader and will meet on a 3-monthly basis in the community at a venue of their choice to access treatment. Stable study participants will be required to have a clinical consultation and VL testing at the facility at 12 and 24 months after enrolment in the study. Each member of the CAG will collect their own 3-month supply of ART from the clinic on this day. Participants who become ill at any stage of the study will report to the clinic as soon as possible. A CAG representative will distribute the drugs to the other CAGs members at the CAG meeting on the same day or the following day. The dates of these visits need to be noted by the facility so that all drugs are pre-dispensed and ready at this visit date.

#### Community ART distribution model by healthcare worker (6-month supply of drug)

Participants enrolled at facilities in this arm will be given a 6 months’ supply of their ARVs at study enrolment. Their next ART refill will be conducted in 6 months in the community at a health outreach or health post (halls, churches or kiosks). These encounters in the community will be on an individual provider-patient basis, not as part of a CAG. The ART refill will be conducted by a healthcare worker who has been appropriately trained and certified for dispensing ART to stable patients. Study participants’ adherence will be assessed at this community distribution point as well as screening for TB, pregnancy, and other common conditions. Patients found to have any conditions that cannot be managed in the community will be referred to the health facility for further follow-up. These participants will continue to receive 6 month refills in the community if they remain stable (i.e. have VL less than 1000 copies per mL). Stable participants will be required to have a clinical consultation and VL done at the facility at 12 months and 24 months after enrolment.

Co-trimoxazole (CPT) and isoniazid (IPT) will also be provided to all arms based on the assigned ART dispensing interval and Lesotho guidelines.

### Study measures and outcomes (data collection matrix)

The schedule of visits and evaluations to be done are outlined see Additional file 1. The primary outcomes to be compared between arms for this study are retention and VL suppression. Secondary outcomes will include the following (i) comparisons in cost effectiveness between arms, (ii) acceptability of models to patients and service providers and (iii) measurable gains in decongestion in facilities through motion time assessments/analysis.

### Entry visit/baseline procedures

For those that enrol, the following detailed data will be collected and recorded from clinical and medical record review: HIV history, HIV-related medications, date of ART initiation, prior adherence/VL data, socio-demographic data including age, gender, level of education, estimated distance and time to travel to clinic, employment status, disclosure status and marital status. Costing data (patient cost data) including travel costs to clinic, opportunity cost of time spent on medication pickup visits etc., will be recorded.

### Viral load testing for enrolled patients (at 12 and 24 months)

As the study will be using VL results from routine monitoring as an inclusion criterion, some participants will have a VL test due soon after enrolment into the study. To synchronize patient care and study VL tests, due routine VL tests will be done as scheduled unless the test is due at 2 months before or after the required study-specific VL test at 12 and 24 months for the study, in which case the study-specific VL will be used for patient management. At 12 and 24 months after enrolment in the study, all patients are required to visit the facility for a VL test.

In this study, there will be no active contact with study participants during the period of follow-up. However, when participants are due for the above-mentioned routine, annual VL assessments, study staff will help ensure each site has capacity to collect these samples and will support systems that help to provide results back to sites. VLs will only be performed on individuals who return for visits and no tracing will be performed by the study for obtaining annual VLs. VLs will be considered within the window for the annual visit if they are performed in a window of +/− 90 days.

### Study end points and definitions

Endpoints will be determined by record reviewing. The primary endpoint will be reached (12 months) and re-assessed after the 24-months. Endpoint data collection will include:

(i) Retention in care defined as participant attrition, where attrition is defined as either death (all-cause) or loss to follow-up (LTFU). LTFU will be defined as no facility or ART collection for > 90 days after the last missed scheduled ART collection. Participants with documented transfers to another clinic will be considered retained at the next immediate time point (12 or 24 months), and will then be censored.
(ii) Suppressed VL of < 1000 copies/mL done as part of annual VL or performed at any other time during the follow-up, if ordered by clinician due to clinical concern (secondary).

The following will be considered not retained unless otherwise noted:

(i) Retained in care but with transitioned off assigned study arm for any reason (patient preference, provider preference, development of ART toxicity requiring switch and closer monitoring and any other complications)
(ii) Transferred to another clinic (if a documented transfer, will be considered retained and analysed as such at the next assessment point.
(iii) Death.

### Management of patients who develop comorbidities and those that become pregnant

Participants who develop TB, other opportunistic infections or comorbidities may require more frequent follow-ups than required by their dispensing interval. If they are in the 3MC or 6MCD arm, they will be considered unstable and are to be followed up at the facility hence transitioned off the study arm. These individuals will remain in observational follow-up but considered not retained for analyses. Providers will make these decisions and the study will track such outcomes. Frequency of ART dispensing will be determined based on national guidelines and clinician assessment. These events will be captured through patient routine records. Women who become pregnant whilst in the study will also require more frequent clinical visits for antenatal and postpartum care than required by their dispensing interval. If they are in the 3MC or 6MCD arm, they will return to be followed up at the facility. Frequency of ART dispensing will be determined based on national guidelines for ART in pregnancy and clinician assessment. These events will be captured through patient routine records. These individuals will remain in observational follow-up.

### Secondary outcomes

#### Time motion analysis

Regarding assessing the gains of facility decongestion, a pre- and 24-month post survey time-flow evaluation of patient waiting times will be conducted. These evaluations will be done over 5 days at each of the 30 facilities. These data will be collected using a study-specific time flow recording sheet for all patients visiting the facility during these 5 days. Mean total visit time, mean waiting time to be seen by the clinician and mean time for drugs to be dispensed will be calculated. Additionally, the monthly number of non-pregnant adults > 18 years ‘newly initiated on ART and receiving ART and provider-initiated counselling and testing at study facilities will be captured from facility records over the study duration to determine trends.

#### Feasibility of MMD of ART in CAGs and community distribution

The feasibility assessment and analysis will include technical, resource and structural aspects. Data will be collected prospectively on study-specific forms at the beginning of the study,12 and 24 months. Before the study activates, facilities will be assessed on their ability to do essential medicine quantifications and forecasting. Each facility’s ability to generate quality data (measured by pre-determined criteria) and to ensure constant supply of medicines to the study participants through efficient ordering and delivery processes will be assessed. Feasibility will also be analysed by the ability of facilities to source adequate storage facilities, utilize efficient transportation systems and the turnaround time for ordering of medicines. For those within CAGs, attendance registers will be used to get a sense of individuals participating rates.

#### Qualitative data collection

Qualitative data will be collected at baseline, 12 and 24 months. Primarily, it will be to explore the acceptability of CAGs and community ART distribution by patients and service providers, patient satisfaction and improvement in quality of care at the facilities. Focus group discussions (FGD) with study participants in the 3MC and 6MCD arms will be held to explore the range of issues regarding acceptability of CAGs and community ART distribution. Each facility will conduct one FGD with up to 10 study participants at each data collection point. A total of 20 FGD will be held at each data collection point. Key informant interviews (KII) will be done with service providers including the facility manager and healthcare workers. Each of the 20 facilities will have a key informant selected for the interviews. A total of 20 KII will be collected for the study at each data collection point.

This will give a total of 40 data points for analysis for the qualitative research. It is hoped that a saturation of findings will be reached by this sample size. Data will be collected by trained facilitators in each FGD using piloted discussion and interview guides for the FGD and the KII respectively. Each FGD will be conducted by 1 facilitator and 1 note taker, whilst KII will be conducted by 1 interviewer. Data collection for FGD will be in Sesotho and it is assumed that all service providers are conversant in English. All qualitative data will be recorded, transcribed, translated to English and coded. All participant in FGDs and KIIs will be required to sign consent to participate and to be recorded.

#### Costing and cost-effectiveness

A micro-costing approach, also known as the bottom-up method will be used. This will allow the tracking of every input in carrying out the study interventions. However, this approach will be supplemented by a macro-costing approach of fixed costs such as infrastructure, building, equipment, utilities etc. To ascertain the cost to provider, we will estimate the fixed and variable costs to care. Fixed cost to patients will include (i) equipment and vehicles (ii) building and Infrastructure and (iii) administration costs and other above point-of-care costs. As for variable costs, these will include but not limited (i) outpatient care (type and number of visits) (ii) Inpatient care (number of bed days in care) (iii) other services (ARTs, laboratory tests, non-ART drugs, consumables etc.).

Patient level costing, also known as direct non-medical costs will also be collected. These costs are borne by either the patient or relatives of the patient to improve the care towards the patient. This study intervention is expected to result in patient level benefits such as reduced cost for care seeking, productivity benefits due to reduced time spent seeking care and informal care effects. Data to assess patient level costs will be collected from a randomly selected sub-sample of patients from each arm. Costing outcomes will include:

(i) average cost per patient retained in care and virally suppressed at 12 and 24 months (cost per patient retained)
(ii) annual cost per patient in each cohort/arm (per patient year cost)
(iii) cost-effectiveness of the intervention arms will be compared with respect to retention in care and viral suppression.

#### Safety assessment, monitoring, and reporting

Participant safety will be carefully assessed, monitored and reported at multiple levels throughout this study. Site investigators, clinical management committee, and an independent data and safety monitoring board will be set up to continuously assess, monitor and report on all safety related matters. All protocol deviations, or unintended consequences/adverse events related to the study and its design will be recorded and appropriately addressed.

#### Statistical analysis, power and sample size considerations

Descriptive data of the study population stratified by treatment arm will presented as medians and interquartile ranges for continuous variables and proportions for categorical variables.

Our analysis will use an intention-to-treat approach in which generalized estimating equations (GEE) will be computed to estimate risk differences and risk ratios and associated 95% confidence interval for the effect of each intervention arm compared to the control (Patients receiving 3 monthly ART supply at facilities), specifying for clustering by facility. A small cluster size variance correction will be used. Should we identify any baseline imbalances between study arms, we will adjust for these through multivariable regression models and report adjusted effect measures and corresponding 95% confidence intervals.

Qualitative analysis will be employed in this study to ascertain feasibility, acceptability of CAGs and community ART distributions by patients and service providers. Summative content analysis which involves counting and comparisons of keywords or content, followed by the interpretation of the underlying context will be conducted. The data will be further analysed for emerging key themes and the findings will be interpreted based on each research question. Quotes will also be extracted for each of the themes that emerge from the data. It is anticipated that MMD will reduce both facility and patient costs of treatment and that it will be cost-saving compared to standard of care. For facilities, fewer clinic visits by patients should save the time of providers and support staff. For patients, fewer clinic visits are expected to reduce the costs of travel and to save time. The study will estimate differences in both provider and patient costs. Cost-effectiveness will be estimated as the average cost per successful outcome (patient retained at 12 and 24 months).

### Sample size estimate

Sample sizes were determined for a cluster randomized non-inferiority trial with retention in care at 12 months as the primary outcome. A total of 30 study sites will be available for randomization, with three study arms, 10 clusters per arm, and participant enrolment numbers will be assumed to be equal at each site. The probability of patient attrition 12 months after study enrolment is assumed to be 5%. An intra-cluster correlation coefficient of 0.01 for patient retention/attrition amongst stable ART patients is assumed from a previous cluster randomized trial including stable ART patients [9]. The non-inferiority limit is specified as 3.25%. Assuming α = 0.05, power of 85%, and using a one-sided Z-test (un-pooled) statistic, approximately 192 enrolled participants per facility will be required with a total sample size of 5760 participants, representing 1920 participants per arm.

### Ethical considerations

All participants will receive information about the study and have opportunity to ask questions. Written informed consent will be obtained from participants after they receive comprehensive details on (i) the purposes of the study; (ii) the allocation process; (iii) the use of data and the means of assuring confidentiality; (iv) voluntary participation and the participant’s right to withdraw from the study at any time and (v) any potential harm that could occur because of the intervention. Consent will be acquired prior to obtaining any study measurements. The study protocol was developed using the Standard Protocol Items: Recommendations for Interventional Trials (SPIRIT) Checklist (see Additional file 1) and adheres to the SPIRIT recommendations. The trial is registered with ClinicalTrials.gov as NCT03438370 and has received ethical clearance from the Lesotho National Health Research Ethics based at the Ministry of Health as well as Chesapeake Institutional Review Board in the USA [Number: ID49–2017 and MOD00231007 respectively].

## Discussion

In this paper, we present and describe a demonstration study protocol that will evaluate retention in care, virologic suppression, feasibility and cost effectiveness of community based differentiated models of MMD approaches of ART among stable HIV patients in Lesotho. Community based models for delivering ART have potential of being an integral part of strengthening the HIV treatment cascade. To meet the UNAIDS targets to end the AIDS epidemic by 2030, a need exists in diversifying ART delivery in ways that are acceptable to communities and maintaining good outcomes including retention and viral suppression [4, 10]. However, community systems must be adequately resourced, including infrastructure that allows for linking and integration with health facilities, allowing for better sustainability and scaling -up.

The WHO has recommended provision of ART through community models, only if operating guidance and monitoring are provided [5]. These strategies are to ultimately significantly reduce the burden experienced by patients and health care providers. Though all models, are to include stable patients, in this context virologically suppressed, and those who are able to adhere to ART [11], the community multi month dispensing approaches including both CAGs and community ART distribution points have been shown and postulated to provide gains for patients by (i) reducing time and transport costs (WHO, 2016), (ii) increasing peer support for adherence (through clubs and CAGs) [11, 12] (iii) reduced defaulting and enhancing community participation [5]. Whereas, for health providers it (i) reduces workload (ii) and maintains and improves health outcomes and improves self-management of patients. Additionally, these approaches, could be extended to key populations including migrants in Sub Saharan Africa, where patients move away from their homes for work and other economic reasons [13]. A study among migrants, assessing healthcare needs, preferences and accessibility barriers of HIV-positive migrant patient populations in high disease burden, borderland districts of Lesotho showed that less than 7% opted for a 1–2 month ARVs refills [14]. Whereas 30.2%and 63% indicated a preference for 3–4 month and 5–6 month refills respectively. More than 65% encountered barriers to receiving their ARVs and about 25% defaulted while abroad [14].

Previous lessons have been learnt to allow for improved management of risk when implementation multi month dispensing ART projects. We anticipate this will include a need for reliable supply of medication [15], access to quality clinical data and linkages, including VLs results, frameworks to support lay worker’s cadre [16, 17], involvement of community stakeholders, and reliable data systems and records of key indicators. The design to be used namely a pragmatic, parallel, cluster-randomized non-inferiority design has the following advantages that add rigor to findings [18]. Additionally, the non-inferiority design aims to determine whether a new approach is not worse than a standard approach by more than a preset margin [19].

In Lesotho, viral load monitoring is performed annually for stable adult’s patients on ART. We anticipate missing viral results. Viral load suppression is thus a secondary outcome. Our findings will be interpreted with care when generalizing to other settings, as there are varying definitions of the term stable patients [20], which is an inclusion criterion in the current study. Additionally, we will not ascertain outcomes of unstable ART patients that will be screened out who may have potentially benefited from the interventions. Large quantities of drugs will be supplied to those that will enroll in the 3 and 6-month arms (including ART, CPT and IPT). This will be challenging for patients who have not disclosed their HIV status to family members leading to erroneous disclosure of HIV status. There is risk for patients to share medication with peers. Both participants and providers will not be blinded, and this might lead to participants wanting to transfer to another arm. Regardless of arm, participants are more likely to be retained if they are aware they are part of a study.

Despite these limitations, this study has a robust design, it is will be the first randomized study to explore the outcomes of retention, virologic suppression and cost effectiveness through differentiated community ART distribution approaches in Lesotho. The study will provide important data regarding the effectiveness of community models for ART distribution and is anticipated to inform policy regarding best practice among HIV-positive patients receiving ART and what is needed to strengthen health systems for rapid scaling up of ART in a contextualized, sustainable and cost-effective manner.

### Trial status

Recruitment and enrolment began 7 August 2017 and is still on going.

## Additional file

Additional file 1: Data collection matrix and schedule of events. (PDF 593 kb)

## Abbreviations

3MC: 3 monthly ART supply through community ART groups
3MF: 3 monthly ART supply at facilities
6MCD: 6 monthly ART supply through community distribution points
AIDS: Acquired immunodeficiency syndrome
ART: Antiretroviral treatment
CAD: Community ART distribution points
CAGs: Community ART groups
CD4: Cluster of differentiation 4
CPT: Co-trimoxazole
FGD: Focus group discussions
GEE: Generalized estimating eqs.
HIV: Human immunodeficiency virus
IPT: Isoniazid
KII: Key informant interviews
LMICs: Low-middle income countries
LTFU: Lost to follow up
MMD: Multi-month dispensing
PEPFAR: U.S Presidential Emergency Plan for AIDS Relief
PLHIV: People living with HIV
SPIRIT: Standard Protocol Items: Recommendations for Interventional Trials
TB: Tuberculosis
UNAIDS: Joint United Nations Programme on HIV and AIDS
USAID: United States Agency for International Development
VL: Viral load
WHO: World Health Organization
