# Supplementary material for: Outcomes of community-based differentiated models of multi-month dispensing of antiretroviral medication among stable HIV-infected patients in Lesotho: a cluster randomised non-inferiority trial protocol
Source: BMC Public Health. 2018 Aug 29;18:1069. doi: 10.1186/s12889-018-5961-0 (PMC6116392; doi:10.1186/s12889-018-5961-0)
Supplement: Supplementary file 1 — Data collection matrix and schedule of events. (PDF 593 kb) [file 12889_2018_5961_MOESM1_ESM.pdf]

Data collection matrix and schedule of events

Evaluation	Entry	12 months	24 months
**Enrollment**			
• Screening	X		
• Informed Consent	X		
• Enrollment	X		
**Interventions**			
• Standard of Care Dispensing (Control)	◆────	────	────◆
• 3 – Month Dispensing	◆────	────	────◆
• 6 – Dispensing	◆────	────	────◆
**Evaluations**			
**Clinical data**<br>• Date HIV diagnosis<br>• ART start date<br>• Medications at entry (ART, CPT, IPT)<br>• ART dispensing interval at time of entry<br>• CD4 at ART initiation/CD4 if documented within 12 months prior to entry<br>• Viral load at entry	X		
**Socio – demographic data**<br>• Age<br>• Marital status<br>• Gender<br>• Education	X		
**Medical record review for endpoint^**<br>• Retention in care*<br>• Viral load<br>• ART regimen changes<br>• Medication review [CPT (Septrin); IPT]<br>• Current dispensing interval (*if changed, date of change and reason for change documented*)<br>• Laboratory tests recorded, if any (CD4)		X	X
**Time and Motion Analysis**<br>• Patient wait times	X		
Cost Assessment	X		
Clinic Resource Allocation Assessment	X	X	X
Focus Group discussions with participants	X	X	X
Qualitative in-depth interviews with service providers	X	X	X

^Individuals who meet an endpoint will be followed for the remainder of the study in observational follow-up;
*Default defined as out of ART ≥90 days

ART: Antiretroviral treatment; CD4: cluster of differentiation 4; CPT: Co-trimoxazole; HIV: human immunodeficiency virus; IPT: isoniazid
